# Supplementary figures and images for: Glucosyltransferase-dependent and independent effects of Clostridioides difficile toxins during infection
Source: PLoS Pathog. 2022 Feb 17;18(2):e1010323. doi: 10.1371/journal.ppat.1010323 (PMC8890742; doi:10.1371/journal.ppat.1010323)

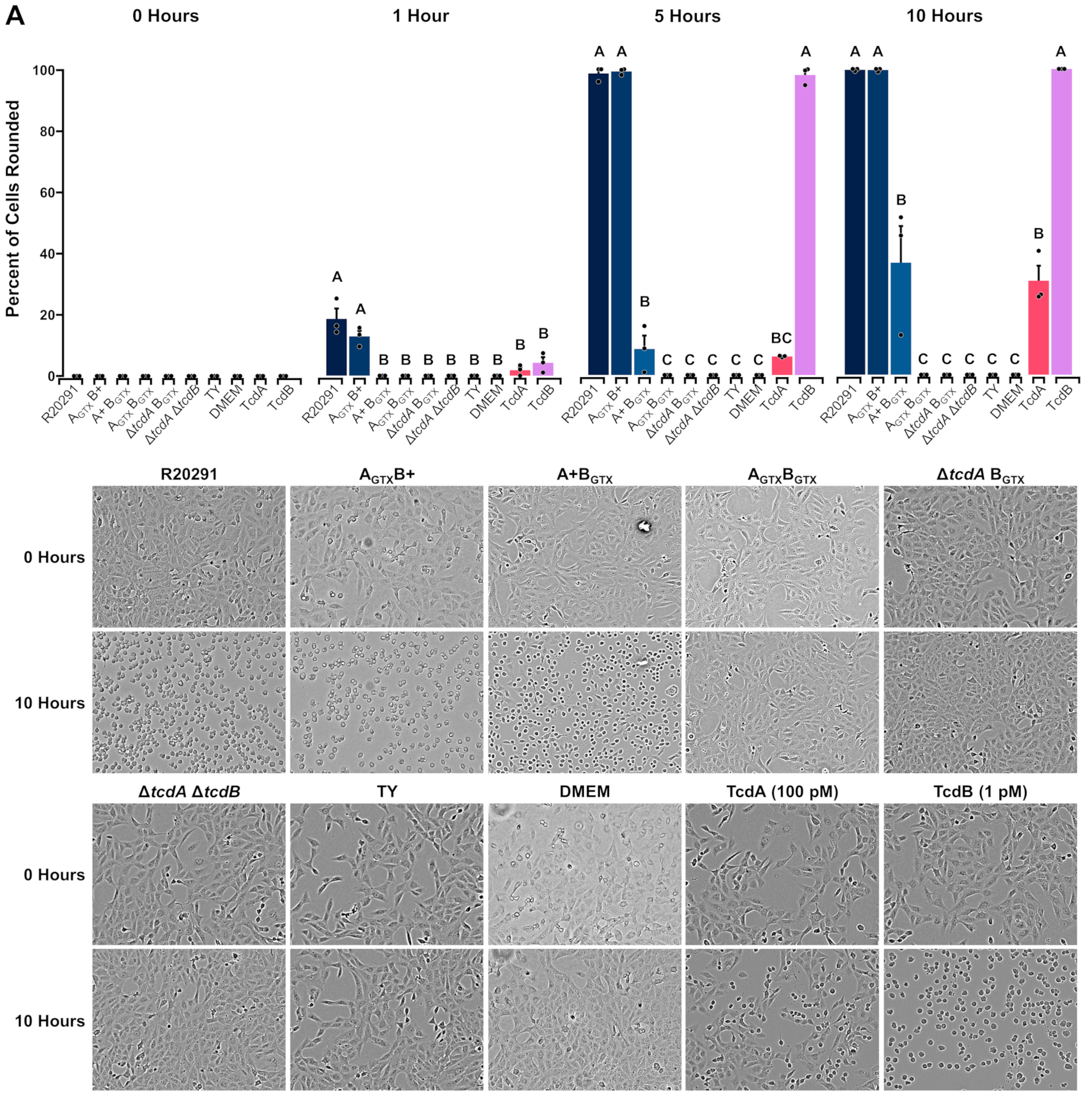

Supplement: S1 Fig — (A & B). Supernatants of C. difficile strains, purified recombinant TcdA (100 pM), TcdB (1 pM), tryptone-yeast (TY) medium, and Dulbecco’s Modified Eagle Medium (DMEM) were used to functionally characterize SNMs and knockout mutations affecting TcdA and/or TcdB in vitro. (A) Each point is the percentage of rounded Vero cells (n = 3 independent experiments) that have undergone toxin-mediated cytopathic changes, and bars represent group means at each hour post-intoxication. Error bars denote standard error of the mean. Significantly different groups as determined by Tukey’s HSD are shown as letters, where groups containing multiple letters are not significantly different to individuals containing those single letters (p < 0.05). (B) Representative images of Vero cells after 0- or 10-hours post-inoculation with each individual treatment. (TIF) [file ppat.1010323.s001.tif]

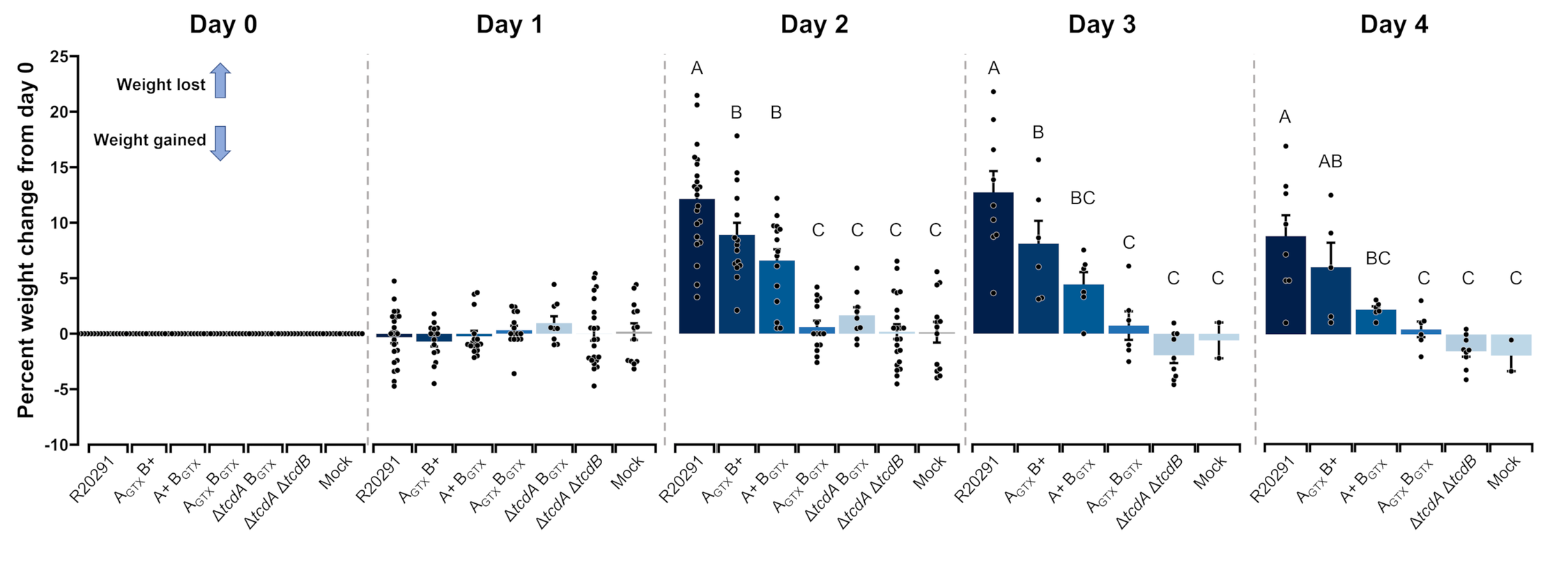

Supplement: S2 Fig — Positive increases signify weight lost and negative decreases indicate weight gained. Each point is an individual animal and bars represent group means at each day. Error bars denote standard error of the mean. Significantly different groups as determined by Tukey’s HSD test are shown using letters where groups containing multiple letters are not significantly different to individuals containing those single letters (p < 0.05). (TIF) [file ppat.1010323.s002.tif]
